# Pleomorphic liposarcoma with liver metastasis diagnosed by combined fine‐needle aspiration cytology and core‐needle biopsy

**DOI:** 10.1002/dc.24875

**Published:** 2021-09-15

**Authors:** Valeria Ciliberti, Pasquale Cretella, Pio Zeppa, Alessandro Caputo

**Affiliations:** ^1^ Department of Advanced Biomedical Sciences, Pathology Unit University of Naples Federico II Naples Italy; ^2^ Department of Medicine and Surgery University of Salerno Salerno Italy

**Keywords:** core‐needle biopsy, fine‐needle cytology, liver metastasis, pleomorphic liposarcoma

## Abstract

Pleomorphic liposarcoma (PLPS) is the rarest liposarcoma subtype, with high‐local recurrence and metastasis rates. Fine‐needle aspiration cytology (FNAC) is successfully used in the diagnosis of primary or metastatic soft tissue tumors, but liver metastases of PLPS diagnosed by FNAC have never been reported. The cytological diagnosis depends on the identification of lipoblasts with sharply defined cytoplasmic vacuoles indenting and distorting the nucleus in the context of a pleomorphic tumor and in a proper clinical and imaging context. Despite its aggressive behavior, hematogenous liver metastases are rare, with just one case reported in literature. A case of PLPS liver metastasis and concomitant primary tumor diagnosed by FNAC and core needle biopsy is herein described.

## 1 INTRODUCTION

The WHO Classification of Soft Tissue and Bone Tumors, 5th Edition^1^, ^2^ classifies different LPS subtypes with different oncogenic drivers, preferred localizations, histological features, and clinical behaviors. Pleomorphic liposarcoma (PLPS) is the least common and most aggressive LPS subtype. PLPS usually arises in the proximal extremities and retroperitoneum and mainly occurs in middle‐aged and elderly individuals. PLPS has a high rate of local recurrence and is largely resistant to conventional chemotherapy. Despite the aggressiveness and the tendency to local recurrences, the rate of hematogenous metastases of PLPS is relatively low (20%), with the most common sites being the lung, pleura, bone, and pancreas.^1^ To the best of our knowledge, only one case of PLPS metastasizing to the liver has been reported to date.^3^ Fine‐needle aspiration cytology (FNAC) is successfully used in the diagnosis of primary or metastatic soft tissue tumors including liposarcoma,^4^ but liver metastases of PLPS diagnosed by FNAC have never been reported. The FNAC features of a PLPS with liver metastases as first clinical evidence are herein described. Both FNAC diagnoses were confirmed by core‐needle biopsy (CB).

## 2 CASE REPORT

**FIGURE 1:**
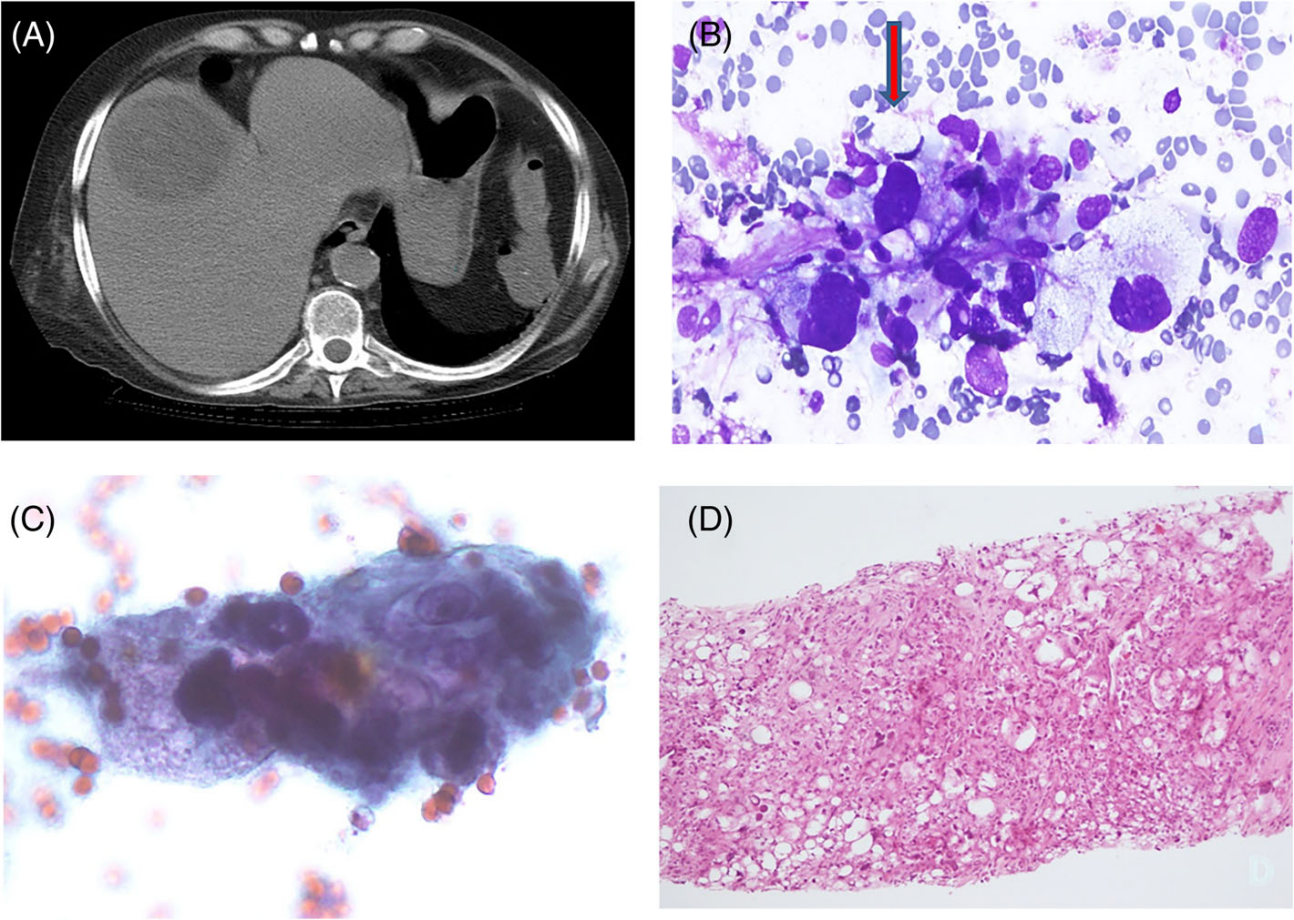
(A) CT scan of a hypodense, well‐circumscribed liver mass. (B) FNAC smear showing pleomorphic cells with vacuolated cytoplasm and focal lipoblastic differentiation (arrow) (Diff‐Quik stain, 430X). (C) A group of epithelioid cells with atypical nuclei and vacuolated cytoplasm (Papanicolaou stain 430X). (D) Core biopsy showing a pleomorphic sarcoma with lipoblastic differentiation (Hematoxylin–Eosin, 270X)

**FIGURE 2:**
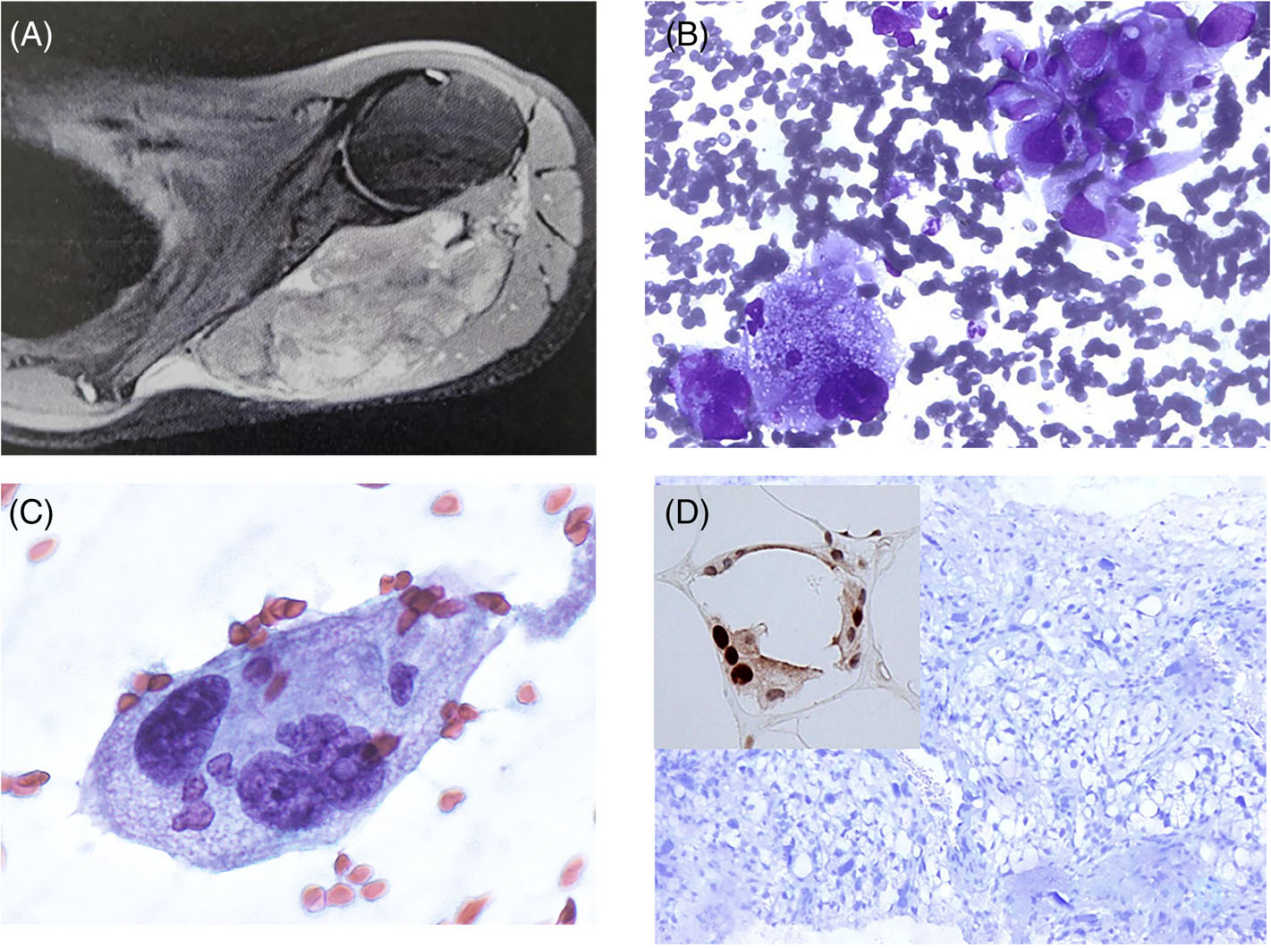
(A) MRI‐scan of the primary PLPS located in the deep soft tissue of the left shoulder girdle. (B) FNAC smear showing pleomorphic cells with vacuolated cytoplasm, similar to the tumoral cells from the liver mass (Diff‐Quik stain, 430X). (C) Isolated, giant multinucleated cell with atypical nuclei and microvacuolated, well defined, cytoplasm (Papanicolaou stain 630X). (D) Negative immunostaining for MDM2 (270X), with positive control (inset) on core biopsy section

A 51‐year‐old man with a history of heavy smoking and lung squamous cell carcinoma 8 years before, presented complaining of general malaise and weakness as well as pain in the right upper abdominal quadrant. Serum and hematological parameters were normal; the patient did not suffer from any hepatopathy but he had undergone lung lobectomy (for a pT1C, N0, M0 squamous cell carcinoma) and six cycles of cisplatin and gemcitabine chemotherapy. Because of his past medical history, a metastasis from the primary lung carcinoma was suspected. The patient underwent abdominal CT and ultrasonography (US), which revealed a 52 × 48 mm, hypoechoic, well‐defined mass located in the right liver lobe (Figure 1A). A diagnostic strategy was conceived with clinicians and accepted by the patient, who signed an informed consent for FNAC and CB, if necessary, and for the use of pathological information and residual diagnostic material for scientific purposes. An US‐guided FNAC was then performed, using a 22G spinal needle: one smear was alcohol‐fixed for Papanicolaou staining and two air‐dried smears were stained with Diff‐Quik. Rapid on‐site evaluation (ROSE) showed epithelioid cells, singly and in groups, with evident nuclear atypia. Large multinucleated cells with atypical nuclei and lipoblasts with sharply defined cytoplasmic vacuoles indenting or distorting the nucleus were observed (Figure 1B). Cytological features were consistent with a poorly differentiated clear‐cell neoplasm. Because of the ROSE data, core biopsy was immediately performed using a 18G × 100 mm HistoCore needle (Germany), inserted in the same trail of the previous FNAC. The resulting 15‐mm–long fragment was fixed in formalin, processed and stained with hematoxylin and eosin. Because of the FNAC/CB diagnosis, a search for the primary tumor was started. A total body MRI scan revealed a 50‐mm mass located in the deep soft tissue of the left shoulder girdle (Figure 2A). Therefore, 7 days after the primary liver FNA/CB, a US‐guided FNAC and subsequent CB were performed on the shoulder tumor, during the same procedure, like previously described for the liver lesion. The cytological and histological features of the soft tissue mass were similar to the corresponding liver tumor and are described below.

## 3 CYTOLOGICAL AND HISTOLOGICAL FINDINGS

FNAC smears and CB histological sections were similar in both the liver and soft tissue lesions. Smears were hypercellular, showing tightly aggregated and loose groups of highly pleomorphic cells with epithelioid features (Figures 1B and 2B), Scattered among these groups, lipoblasts could be observed with both micro‐ and macro‐vesicular fat droplets (Figures 1C and 2B). At higher magnification, cells showed scalloped nuclei with coarse chromatin and large nucleoli (Figures 1B and 2C). Large, multinucleated cells with clear cytoplasm and two or more atypical nuclei were also present (Figures 2C). Cytological features were consistent with a pleomorphic sarcoma with liposarcoma differentiation. Histologically, the liver metastasis and primary tumor both showed nodular or sheet‐like arrangements (Figures 1D). Tumoral cells showed different cytological features, ranging from epithelioid to pleomorphic. The epithelioid cells showed oval to round nuclei and abundant clear to slightly eosinophilic cytoplasm; the cells with lipoblastic differentiation showed hyperchromatic and scalloped nuclei and multivacuolated (rarely uni‐vacuolated) cytoplasm. Pleomorphic and spindle cells were the third component, with the latter having a vaguely fascicular pattern. Areas of necrosis and hemorrhage were also present. The finding of pleomorphic lipoblasts in the liver mass favored a pleomorphic liposarcoma, and, despite there being a report of a single primary liver PLPS,^5^ a metastatic tumor was more likely. Immunohistochemistry (IHC) was performed on additional sections and showed positivity for vimentin and CD10 and negativity for AE1/AE3, Hep‐Par1, and MDM2 (Figure 2D). According to the clinical, cytological, histological and IHC data, a diagnosis of primary PLPS of the shoulder girdle with liver metastasis was performed. After the diagnosis, a possible genetic background was suspected, despite the negative family history. The patient was referred to a hospital specialized for the diagnosis and treatment of soft tissue tumors, in which the diagnosis of PLPS was confirmed and a genetic basis was excluded. The patient underwent surgical treatment of the primary tumor and stereotactic body radiation therapy (26 Gy at 80% isodose) for the hepatic metastasis and died 8 months later due to liver failure.

## 4 DISCUSSION

The liver is a rare site for PLPS metastases: to the best of our knowledge, only one such case has been described to date.^3^ The present case was challenging because it might have been mistaken for other pleomorphic clear cell tumors. In fact, PLPS usually manifests as a painless mass located in the deep soft tissue and may go unnoticed until it reaches a significant size or metastasizes—as in the present case, in which the liver metastasis was the first evidence of neoplasm. Lastly, in this case, the whole diagnostic procedure was performed with FNAC and CB. The number of studies confirming the effectiveness of FNAC in the diagnosis of adipose tissue neoplasms is increasing,^6^, ^7^, ^8^, ^9^, ^10^ but PLPS requires an accurate histological evaluation. For this purpose, we routinely prepare cellblocks for soft tissue tumors and, in selected cases (solid masses, larger than 20 mm, approachable by US guidance), with the approval of the clinicians and the consent of the patient, we also perform CB for standard histological processing. This case showed all these conditions and ROSE indicated the need for a CB. This sequence of events was similar in both the diagnostic procedures for the liver and shoulder masses. The differential diagnosis included clear cell hepatocellular carcinoma, clear cell cholangiocarcinoma, metastatic renal or adrenal cortical carcinoma, epithelioid angiomyolipoma, and high‐grade sarcoma with no specific differentiation. Hepatocarcinoma was excluded because of the lack of any underlying hepatopathy and by the negativity for AE1/AE3 and Hep‐Par1 at IHC; cholangiocarcinoma was excluded because of the negativity for AE1/AE3 and lack of any ductal differentiation; for the same reasons a possible metastasis from the former lung neoplasm was excluded. Metastatic renal clear cell carcinoma and adrenal cortical carcinoma, again were excluded by the negativity for AE1/AE3 and because of the lack of any renal or adrenal lesion. Epithelioid angiomyolipoma and high‐grade sarcoma with no specific differentiation were excluded by the lack of a predominant vascular pattern and the presence of pleomorphic lipoblasts respectively. Therefore, the diagnosis of PLPS was performed on the basis of the sole microscopic and clinical data, being the vimentin and CD10 positivity unspecific and MDM2 negative (Figure 2D). In fact, while other subtypes of liposarcoma are associated with specific IHC and genetic alterations involving MDM2/CDK4 and DDIT3,^11^, ^12^ PLPS has complex numerical chromosome aberrations and does not express specific markers^12^; therefore, the diagnosis relies on microscopic features only. Finally, even though a primary liver PLPS has been reported once,^5^ the discovery of the primary shoulder tumor and the morphological similarities between the two tumors favored the diagnosis of liver metastasis from PLPS. CB and FNAC are often considered alternative methods, while in this case were conveniently used together offering the synergic advantage of ROSE, cytological features and histological controls at the same time. In conclusion, FNAC could be an important noninvasive procedure for the diagnosis of PLPS in unusual presentations, including the identification of primary tumors.

## CONFLICT OF INTEREST

The authors declare that they have no conflict of interest.

## ETHICS STATEMENT

Written informed consent was obtained from the patient for publication of this article. All potentially identifying information has been anonymized as much as possible. The authors have conducted their study in accordance with the World Medical Association Declaration of Helsinki.

## Data Availability

Data sharing is not applicable to this article as no new data were created or analyzed in this study.
